# Elevated levels of mitochondrial CoQ_10_ induce ROS-mediated apoptosis in pancreatic cancer

**DOI:** 10.1038/s41598-021-84852-z

**Published:** 2021-03-11

**Authors:** Tulin Dadali, Anne R. Diers, Shiva Kazerounian, Senthil K. Muthuswamy, Pallavi Awate, Ryan Ng, Saie Mogre, Carrie Spencer, Katerina Krumova, Hannah E. Rockwell, Justice McDaniel, Emily Y. Chen, Fei Gao, Karl T. Diedrich, Vijetha Vemulapalli, Leonardo O. Rodrigues, Viatcheslav R. Akmaev, Khampaseuth Thapa, Manuel Hidalgo, Arindam Bose, Vivek K. Vishnudas, A. James Moser, Elder Granger, Michael A. Kiebish, Stephane Gesta, Niven R. Narain, Rangaprasad Sarangarajan

**Affiliations:** 1 BERG LLC, 500 Old Connecticut Path, Bldg B, 3rd Floor, Framingham, MA 01710 USA; 2 Department of Medicine, Cancer Research Institute, Beth Israel Deaconess Medical Center, Harvard Medical School, Boston, MA 02215 USA

**Keywords:** Cancer metabolism, Cancer

## Abstract

Reactive oxygen species (ROS) are implicated in triggering cell signalling events and pathways to promote and maintain tumorigenicity. Chemotherapy and radiation can induce ROS to elicit cell death allows for targeting ROS pathways for effective anti-cancer therapeutics. Coenzyme Q_10_ is a critical cofactor in the electron transport chain with complex biological functions that extend beyond mitochondrial respiration. This study demonstrates that delivery of oxidized Coenzyme Q_10_ (ubidecarenone) to increase mitochondrial Q-pool is associated with an increase in ROS generation, effectuating anti-cancer effects in a pancreatic cancer model. Consequent activation of cell death was observed in vitro in pancreatic cancer cells, and both human patient-derived organoids and tumour xenografts. The study is a first to demonstrate the effectiveness of oxidized ubidecarenone in targeting mitochondrial function resulting in an anti-cancer effect. Furthermore, these findings support the clinical development of proprietary formulation, BPM31510, for treatment of cancers with high ROS burden with potential sensitivity to ubidecarenone.

## Introduction

Dysregulated metabolism is considered a hallmark of cancer and is a consequence of adaptive responses to support unregulated proliferation. The Warburg Effect is one such adaptive response characterized by glycolysis addiction for rapid non-mitochondrial ATP synthesis. This phenomenon further supports an increase in cancer cell biosynthetic programs and maintenance of redox balance during uncontrolled proliferation^1^. A potential advantage of aerobic glycolysis in cancer is the ability of cancer cells to reduce generation of mitochondrial derived reactive oxygen species (ROS) to levels advantageous to support survival and proliferation. In fact, strategies aimed at elevating ROS burden in cancer has been investigated as an attractive therapeutic approach to elicit an anti-cancer effect^2^.

Coenzyme Q_10_ (CoQ_10_) is a key component of the mitochondrial electron transport chain (ETC)^3^ and plays a critical role in mitochondrial redox potential and energy production^4^. It is an ubiquitous molecule that exists in all subcellular membranes and recently has been recognized to have more complex biological functions than initially proposed^5^. In addition to its role in ETC function, CoQ_10_ has phenolic antioxidant activity via its ability to undergo hydrogen abstraction by free radicals^6^. Paradoxically, CoQ_10_ also exhibits pro-oxidant activity that occurs either due to a CoQ_10_ semiquinone reaction^5^ or due to a reaction with oxygen when CoQ_10_ is in its oxidized state^7^. In the mitochondria, 40–50% of CoQ_10_ lies in the inner membrane and exists in one of three states: fully oxidized (ubiquinone, also known as Q or ubidecarenone), partially reduced (semiubiquinone), and fully reduced (ubiquinol, CoQH_2_)^8^. In its fully oxidized state, CoQ_10_ accepts electrons from NADH-ubiquinone reductase (Complex I) and succinate-Q oxidoreductase (Complex II), and transfers them to Complex III. CoQ_10_ also distributes electrons between various dehydrogenases, including mitochondrial glycerol 3-phosphate dehydrogenase (mGPDH), electron-transferring flavoprotein (ETF:Q oxidoreductase, ETF:QOR), and dihydro-orotate dehydrogenase (DHODH)^8–10^.

Moreover, studies using isolated mitochondria from rat skeletal muscle found that ROS production occurred at 11 sites associated with substrate oxidation and oxidative phosphorylation^9^. Five of the 11 substrates associated with ROS production were connected to the Q pool, including substrates associated with DHODH, ETF:QOR, mGPDH, and Complex II activity^9^. Under normal physiological conditions, substrates that feed into mitochondrial Complexes I and II (site of CoQ10 binding and activity) produce low levels of ROS that are essential for intracellular signalling^9,11^. Taken together, data support a central role for CoQ_10_ in mitochondrial substrate utilization and generation of ROS; thus, increasing the ROS burden in cancer should be associated with anti-cancer effect.

Although the biological benefits of CoQ_10_ including its anti-cancer potential has been recognized, its therapeutic utility has been hindered by (a) dearth in understanding the significance of anti-cancer potential of oxidized CoQ_10_ and (b) formulation for delivery of oxidized CoQ_10_ to the target tissues and specifically to the mitochondria. BPM31510 is a ubidecarenone-lipid conjugate nanodispersion optimized for delivery of supraphysiologic concentrations of ubidecarenone in cells and tissues, preferentially to the mitochondria. At present, BPM31510 is in clinical trials for solid tumours that exhibit a high glycolytic phenotype including pancreatic cancer and glioma. Although clinical assessment of BPM31510 is underway, the effects of cellular delivery of CoQ_10_ (via BPM31510) in intact cells and tissues has not yet been described. The goal of the study was to define the mechanism by which supraphysiologic levels of CoQ_10_ regulate cell fate, particularly in a cancer model system. Based on the established ability of CoQ10 in ROS generation, it was hypothesized that excess CoQ10 will enhance mitochondrial ROS production and activate cell death in cancer cells. Pancreatic cancers are highly glycolytic^12^ and serve as a good model system to test the hypothesis of the ability of CoQ10 to induce ROS and activate apoptosis in eliciting an anticancer effect. Collectively, the role of CoQ_10_ in substrate utilization and generation of ROS as well as the function of BPM31510 is a unique and novel therapeutic pathway to elicit an anti-cancer effect in metabolically active tumors.

## Results

### Delivery of supraphysiologic levels of ubidecarenone via BPM31510 decreases oxygen consumption rates (OCR) in pancreatic cancer

**Figure 1:**
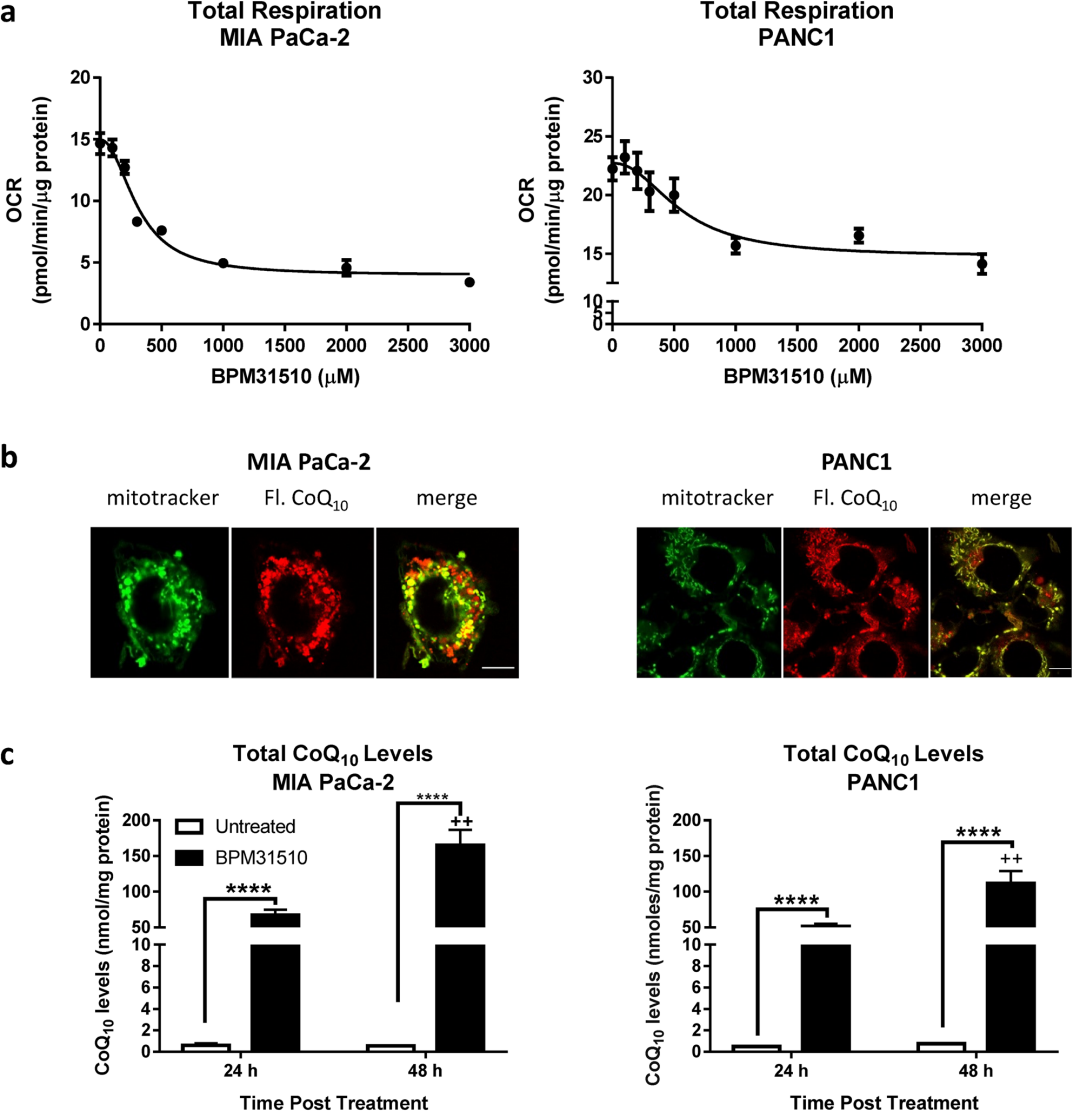
Delivery of CoQ10 into mitochondria by BPM31510 affects total respiration in MIA PaCa-2 and PANC1. (**a**) Dose effect of BPM31510 on total respiration in MIA PaCa-2 and PANC1 cells. State 3 respiration on pyruvate/malate + succinate + glycerol-3-phosphate of cells exposed to BPM31510 at 24 h. The data represent the means ± SEM of three independent experiments with five replicates (n = 15). (**b**) Fluorescently tagged CoQ_10_ (red) from BPM31510 (200 μM) colocalized with the mitochondria (MitoTracker; green) in MIA PaCa-2 and PANC1 cells after 24 h exposure. (**c**) BPM31510 treatment increased the total CoQ_10_ levels in MIA PaCa-2 and PANC1 cells as measured by LC MS/MS after 24 h and 48 h exposures. The data represent the means + SEM of total protein (nmol/mg) from six independent experiments. The data were analysed by Student’s t tests; ****=*p* < 0.0001 compared to untreated control; ^++^=*p* < 0.01 compared to the 24 h time point. The scale bar present in each merged image represents 10 mM.

Altering the dynamics of the Q pool and examining the functional consequence of exogenous CoQ_10_ has been a challenge due to poor intracellular delivery. Thus, the ability of BPM31510 to affect mitochondrial function as a consequence of CoQ_10_ accumulation in mitochondria in pancreatic cancer model was assessed. BPM31510 treatment was associated with a decrease in oxygen consumption rates in MIA PaCa-2 and PANC1 pancreatic cancer cell lines in a dose-dependent manner (Fig. 1a). A formulation of BPM31510 consisting of a fluorescent analogue of CoQ_10_ was used to visualize the uptake and mitochondrial localization in cancer cells using confocal microscopy. In the pancreatic cancer cell lines, fluorescent ubidecarenone accumulated in the mitochondria as indicated by its colocalization with the mitochondria marker MitoTracker (Fig. 1b). CoQ_10_ accumulation occurred in a time-dependent manner: at 24 h, a > 100-fold increase and at 48 h > 150-fold increase over endogenous levels was observed in MIA PaCa-2 and PANC1 cells (Fig. 1c). Importantly, while CoQ_10_ was distributed within various cellular compartments, the greatest enrichment was detected in the mitochondrial fractions (Supplemental Table 1). Together, the data demonstrate that BPM31510 delivers supraphysiologic levels of CoQ_10_ into the mitochondrial Q-pool, and the increase in mitochondrial CoQ_10_ is associated with dose-dependent decrease in oxygen consumption rates.

### Ubidecarenone selectively disrupts substrate-dependent respiration in the mitochondria in pancreatic cancer cells

**Figure 2:**
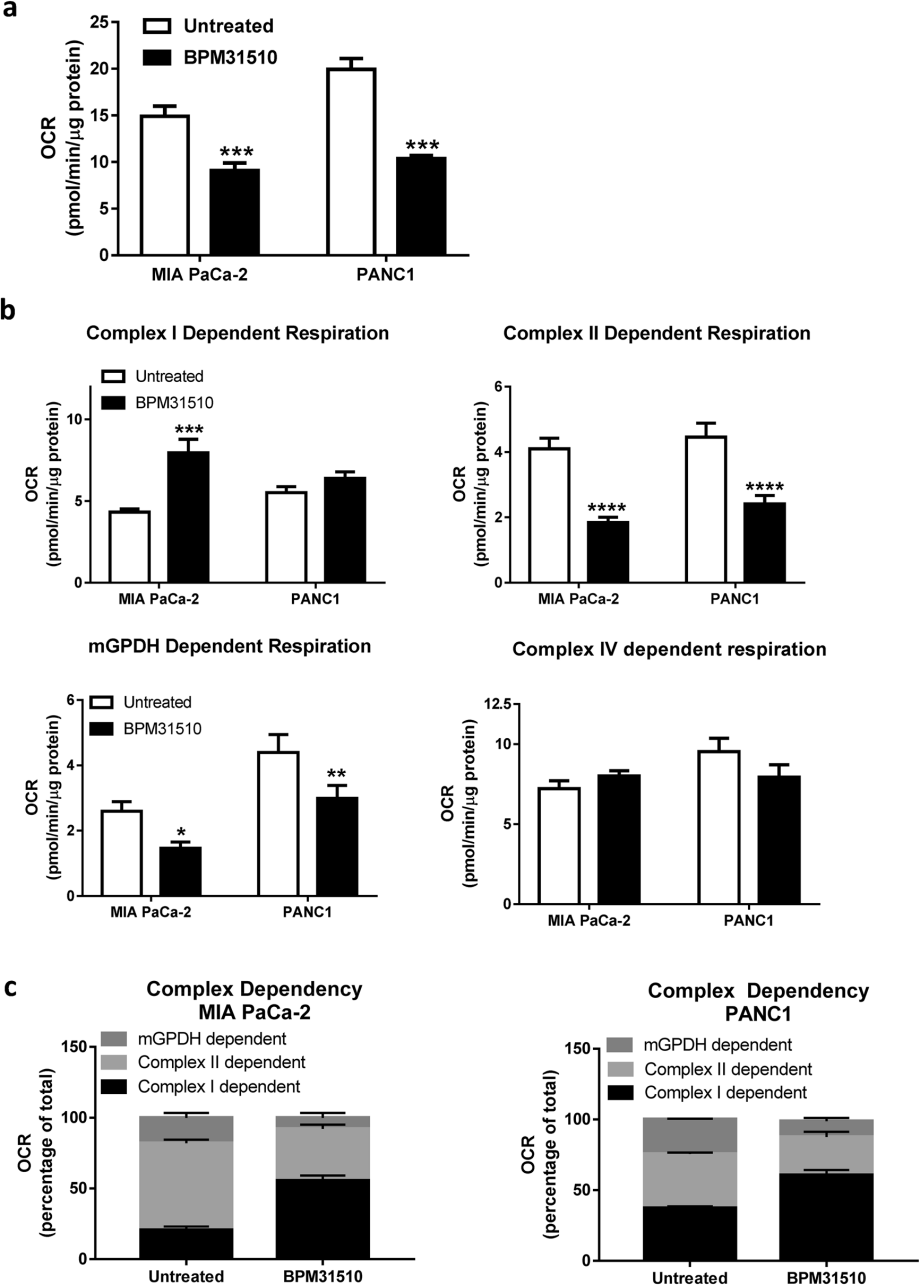
BPM31510 decreases Q-pool linked Complex II and mitochondrial glycerol-3-phosphate dependent respiration, resulting in shifts in respiratory dependency of specific mitochondrial complexes. (**a**) State 3 respiration on pyruvate/malate + succinate + glycerol-3-phosphate in MIA PaCa-2 and PANC1 cells after BPM31510 treatment. (**b**) Effects of BPM31510 on electron transport chain complex (substrate-specific) respiration in MIA PaCa-2 and PANC1 cells. The data represent the means + SEM of three independent experiments. (**c**) BPM31510 shifts respiratory dependency in MIA PaCa-2 and PANC1 cells. Complex-specific respiration was assessed as described in the Methods section. The data report the percentage of total respiration. The data represent the means + SEM of three independent experiments. The data were analysed by Student’s t-tests; ***p < 0.001 compared to untreated cells.

Next, the effect of BPM31510 mediated increases in the mitochondrial Q pool on specific substrate-driven respiration was assessed in MIA PaCa-2 and PANC1 cells. In these experiments, a BPM31510 dose that resulted in a 50% decrease in OCR for each respective cell line (based on Fig. 1a) was used. As expected, BPM31510 treatment at this dose induced a significant decrease in OCR (Fig. 2a), suggestive of a disruption of the mitochondrial ETC function. Substrate specific respiration was characterized to determine the site of ubidecarenone-induced disruption within the ETC. While we noted differential effects of BPM31510 between cell lines in pyruvate (Complex I)-driven respiration, BPM31510 significantly decreased succinate (Complex II)-driven and mGPDH-driven respiration and had no effect on Complex IV-driven responses in MIA PaCa-2 and PANC1 cells (Fig. 2b).

Previous research indicates that individual mitochondrial respiratory complexes are mutually linked, and compensatory responses can be engaged to manage changes in substrate availability, metabolic flux, and ETC component function to meet bioenergetic demands^13^. Therefore, BPM31510-induced changes in succinate/glycerol-3-phosphate-driven respiration were assessed to determine if it was sufficient to promote compensatory respiratory responses. Oxygen consumption in response to treatment with respiratory complex inhibitors, such as rotenone (Complex I), TTFA (Complex II), or iGP1 (mGPDH), to determine which sites supported mitochondrial respiration were measured. Although MIA PaCa-2 and PANC1 cells exhibited different dependencies under basal conditions, both cell lines increased reliance on Complex I-dependent oxygen consumption after BPM31510 treatment, with a smaller percentage of total oxygen consumption supported by Complex II and mGPDH (Fig. 2c). Conjointly, these data indicate that supraphysiologic increases in mitochondrial CoQ_10_ levels by delivery of ubidecarenone alter mitochondrial respiration by impairing Q-pool dependent Complex II–III-driven respiration.

### Ubidecarenone enhances succinate-dependent and glycerol-3-phosphate-dependent ROS generation, mitochondrial membrane depolarization, and regulated cell death

**Figure 3:**
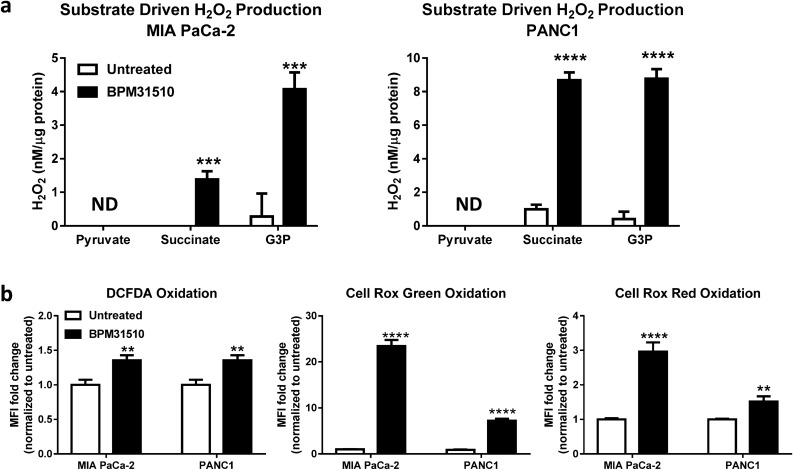
BPM31510 induces oxidative stress via generation of mitochondrial reactive oxygen species (ROS) in MIA PaCa-2 and PANC1 cells. (**a**) Mitochondrial H_2_O_2_ generation during complex I, complex II and GPDH-driven respiration in cells were after treatment with BPM31510 (EC_50_) for 24 h. The data represent the means + SEM of three independent experiments. The data were analysed by Student’s t test; ***p < 0.001 and ****p < 0.0001 compared to untreated. UD = undetected (**b**) Oxidation of multiple redox probes, indicative of oxidative stress, in MIA PaCa-2 and PANC1 cells after treatment with BPM31510 (EC_50_) for 24 h. The data represent the means + SEM of three independent experiments and report the fold change in mean fluorescence intensity (MFI) normalized to untreated controls. The data were analysed by Student’s t test; **p < 0.01 and ****p < 0.0001 compared to the untreated group.

Impairments to the mitochondrial ETC, in particular the loss of efficiency in electron transfer from Complex III or mGPDH to oxygen (as reported above), can result in electron leakage and ROS generation^10^. If unresolved, ROS accumulation promotes oxidative stress, cellular dysregulation, and subsequent cell death^14^. To examine whether BPM31510-dependent impairment of substrate-driven respiration results in concomitant electron leakage and ROS production, a method was established to measure substrate-dependent H_2_O_2_ production by initiating respiration with specific substrates and kinetically quantifying H_2_O_2_ production using Amplex Red reagent (Supplemental Fig. 1). In both control and BPM31510-treated MIA PaCa-2 and PANC1 cells, pyruvate (Complex I)-specific H_2_O_2_ production was minimal, indicating that Complex I is an unlikely site for the exacerbated ROS production observed after BPM31510 treatment (Fig. 3a, Supplemental Fig. 1).

However, in both cell lines, BPM31510 treatment significantly enhanced H_2_O_2_ production that was promoted by succinate and glycerol-3-phosphate. The data suggest that increased ROS production due to BPM31510 treatment arise from the mitochondrial complexes and dehydrogenases with a functional connection to the Q-pool, specifically from Complex II and mGPDH. Furthermore, increased mitochondrial H_2_O_2_ production in MIA PaCa-2 and PANC1 cells following BPM31510 treatment was sufficient to disrupt the cellular redox balance as evidenced by increased oxidation of three distinct cellular redox probes (DCFDA, CellRox Green, and CellRox Red) (Fig. 3b). In all, the data demonstrate that supraphysiologic increases in mitochondrial ubidecarenone levels is sufficient to alter redox homeostasis as well as promote site-specific ROS production at the level of the mitochondria at Complex II and mGPDH.

**Figure 4:**
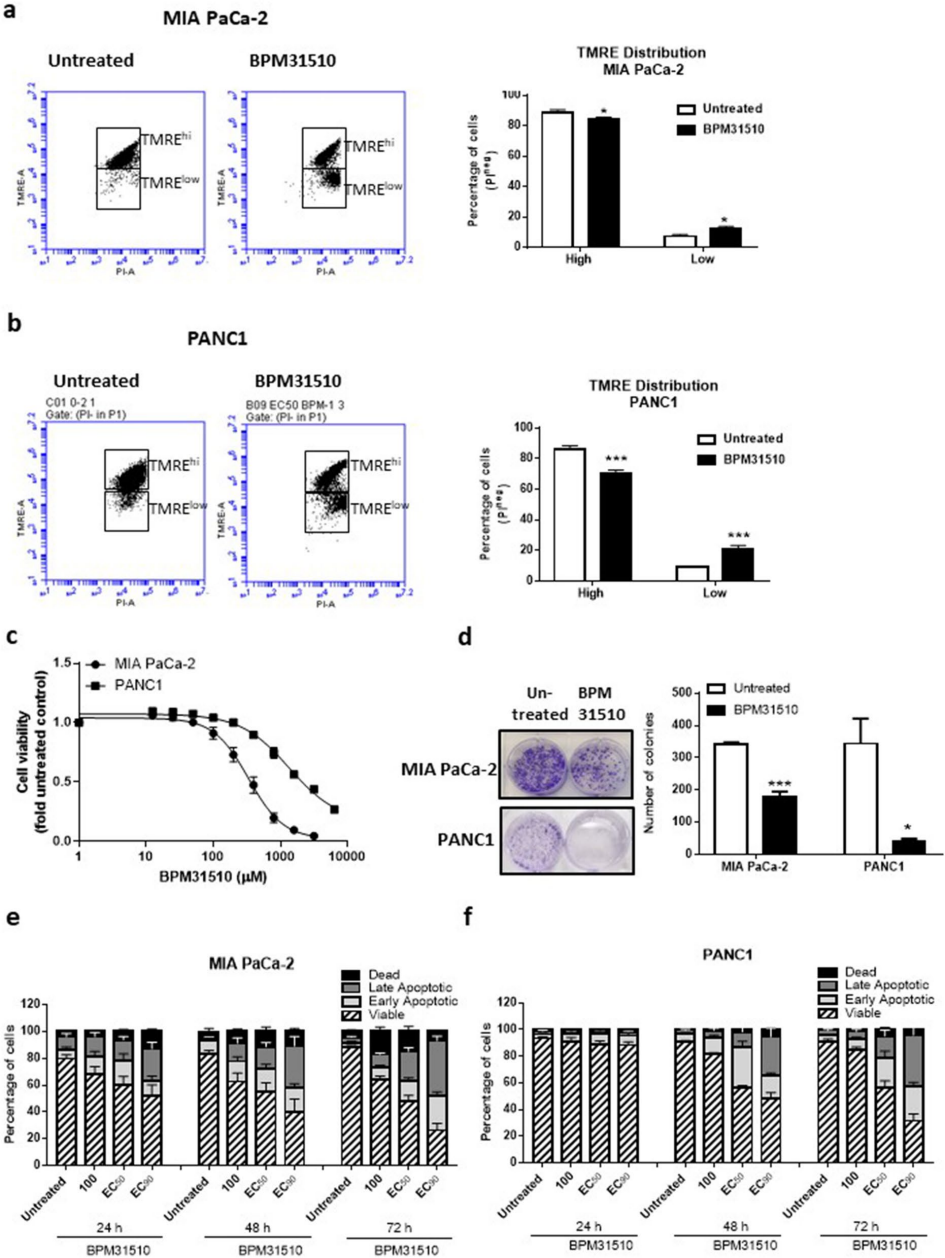
BPM31510 treatment hyperpolarizes the mitochondrial membrane potential prior to promoting regulated cell death in MIA PaCa-2 and PANC1 cells. (**a**) Representative scatter plots of untreated and BPM31510-treated MIA PaCa-2 cells illustrating the gating strategy used to measure the percentage and mean fluorescent intensity of tetramethylrhodamine, ethyl ester (TMRE) high and low cells (Right panel). Percentage of TMRE high-expressing and low-expressing MIA PaCa-2 cells 48 h after BPM31510 exposure. The data represent the means + SEM of three independent experiments. (**b**) Representative scatter plots of untreated and BPM31510-treated PANC1 (Right panel). Percentage of TMRE high-expressing and low-expressing PANC1 cells 48 h after BPM31510 exposure. The data represent the means + SEM of three independent experiments. (Left panel) (**c**) Dose–response of BPM31510 on cell viability in MIA PaCa-2 and PANC1 cancer cells. The data represent the means ± SEM of three independent experiments. (**d**) Colony formation after BPM31510 exposure (EC_50_) for 72 h. Representative images of colonies and corresponding quantitation are shown. The data represent the means + SEM of three independent experiments. (**e**,**f**) Dose- and time-assessment of BPM31510 on Annexin V and propidium iodide (PI) staining in MIA PaCa-2 (**e)** and PANC1 cells (**f**). The data represent the means + SEM of three independent experiments. The data report the percentage of the population that is viable (Annexin V negative and PI negative), early apoptotic (Annexin V positive and PI negative), late apoptotic (Annexin V positive and PI positive), and dead/necrotic cells (Annexin V negative and PI positive). All data were analysed by Student’s t test; *p < 0.05 and ***p < 0.001 compared to the untreated group.

The ability of ubidecarenone to disrupt mitochondrial substrate-dependent respiration and induce ROS production suggests mitochondrial dysfunction in the cancer cells. To assess the functional status of mitochondria after BPM31510 treatment in these cell lines, mitochondrial membrane potential (ΔψΜ) was assessed using the lipophilic cation fluorescent dye TMRE, which is sequestered in active (polarized) but not in inactive (depolarized) mitochondria^15^. The viable [propidium iodide (PI)-negative] cells were subgated into TMRE^high-^ and TMRE^low^-stained populations (Fig. 4 and Supplemental Fig. 3) and found that BPM31510 treatment led to a significant decrease in the percentage of the TMRE^high^ population and a simultaneous increase in the TMRE^low^ population (Fig. 4a,b in MIA PaCa-2 and PANC1 cells, respectively). This demonstrates that mitochondrial depolarization in response to BPM31510 treatment occurs downstream of changes in mitochondrial respiration and ROS production.

Alterations in mitochondrial membrane potential direct the activation of mitochondrial cell death signalling pathways^16^. As cancer cells are in a persistent state of oxidative stress with high levels of ROS production, they have a low tolerance thresholds for additional ROS and activation of cell death pathways^17^. Treatment of both pancreatic cancer cells lines with BPM31510 at doses that altered mitochondrial respiration, redox homeostasis, and ΔψM was associated with a decrease in cell viability (Fig. 4a–c). Consistent with the decrease in cell viability, BPM31510 decreased colony formation (Fig. 4d). There was a significant decrease in the percentage of viable MIA PaCa-2 and PANC1 cells (Annexin V^neg^ and PI^neg^ staining), with a concomitant increase in the percentage of total non-viable Annexin V^pos^ cells (Fig. 4e,f, respectively). Altogether, these data demonstrate that supraphysiologic increases in the mitochondrial Q pool induce regulated cell death in pancreatic cancer cells in vitro.

### Functional complex II and GPDH are sufficient for ubidecarenone-induced ROS production and cell death

**Figure 5:**
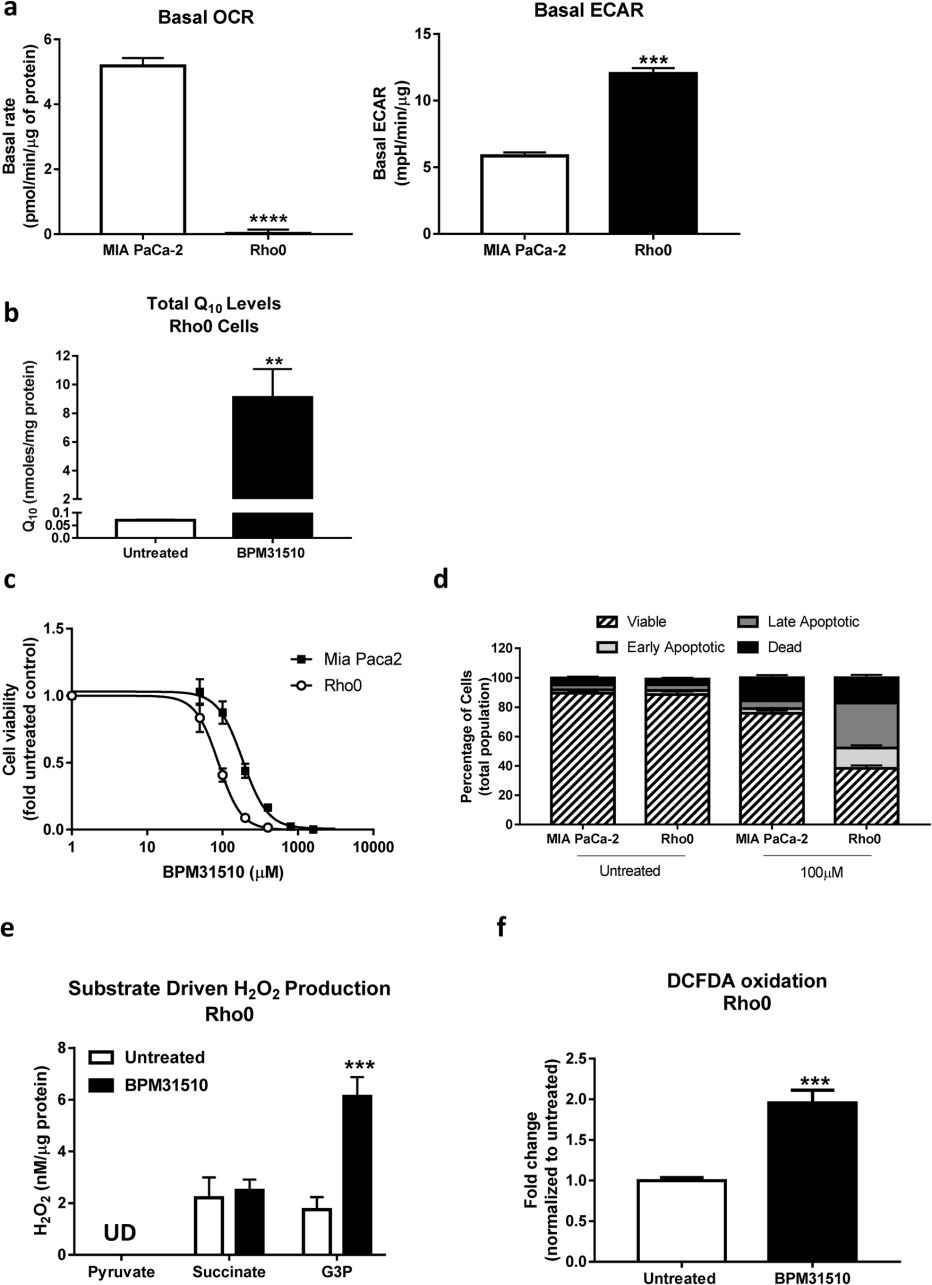
Rho0 cells are more sensitive to BPM31510 than MIA PaCa-2 parental cell lines. (**a**) Mitochondrial DNA depletion in MIA PaCa-2 cells (Rho0) results in very low oxygen consumption rate (OCR) and elevated glycolytic activity. ECAR, extracellular acidification rate. The data represent the means + SEM of three independent experiments. (**b**) CoQ_10_ levels in Rho0 cells after BPM31510 treatment measured using LC/MS/MS 24 h after BPM31510 exposure. The data represent the means + SEM of six independent experiments. (**c**) Dose effect of BPM31510 on cell viability. (**d**) Annexin V and propidium iodide (PI) staining followed by flow cytometry. The data represent the means ± SEM of three independent experiments. The data in (**d**) report the percentage of the population that was viable (Annexin V^neg^, PI^neg^), early apoptotic (Annexin V^pos^, PI^neg^), late apoptotic (Annexin V^pos^, PI^pos^), and dead/necrotic cells (Annexin V^neg^, PI^pos^). (**e**) BPM31510 promotes the generation of mitochondrial H_2_O_2_ during G3PDH-driven respiration in Rho0 cells as assessed by Amplex Red assay. UD = undetected. The data represent the means + SEM of three independent experiments and were analysed by Student’s t test; ***p < 0.001 compared to the untreated group. (**f**) BPM31510 promotes cellular reactive oxygen species (ROS) production in Rho0 cells. The data represent the means + SEM of three independent experiments and report the fold change normalized to the untreated control. The data were analysed by Student’s t test; **p < 0.01 and ***p < 0.001 compared to the untreated control.

As BPM31510 treatment affects the Q-pool between Complexes II and III, its effect in Rho0 cells was examined. Rho0 cells are depleted of mitochondrial (mt) DNA, and thus exhibit functional impairments in Complexes I, III, IV, and V (protein subunits some of which are encoded by mtDNA), resulting in a loss of mitochondrial respiration^18^. However, Complex II and GPDH are functional in Rho0 cells as they are fully encoded by nuclear DNA^19^. Therefore, the Rho0 cells constitute an ideal model to study the requirement of Complex II and GPDH in site-specific disruption of the ETC by ubidecarenone. As expected, compared to parental MIA PaCa-2 cells, MIA PaCa-2 Rho0 cells displayed minimal basal respiration and higher levels of extracellular acidification, suggesting increased glycolytic activity in the absence of functional mitochondria (Fig. 5a). In addition, CoQ10 levels were higher in BPM31510 treated cells, compared to control, indicating that BPM31510 effectively delivered CoQ_10_ to these cells (Fig. 5b).

Interestingly, Rho0 cells were more sensitive to BPM31510-induced cell death than parental MIA PaCa-2, as indicated by a lower IC_50_ (88.1 µM vs. 184.8 µM) for cell viability (Fig. 5c) and an increase in the percentage of the total Annexin V^pos^ population (Fig. 5d). Also, consistent with earlier experiments in parental cell lines, BPM31510 treatment significantly increased G3P-driven ROS production (Fig. 5e), which was substantiated by an increase in total cellular pro-oxidant levels (Fig. 5f). Thus, these data indicate that functional Complex II and GPDH are sufficient for ROS production and subsequent induction of regulated cell death induced by supraphysiologic increases in the mitochondrial Q pool. This finding further supports that the mechanism of cell death is independent of mitochondrial respiration.

### Ubidecarenone affects tumour growth in vivo

**Figure 6:**
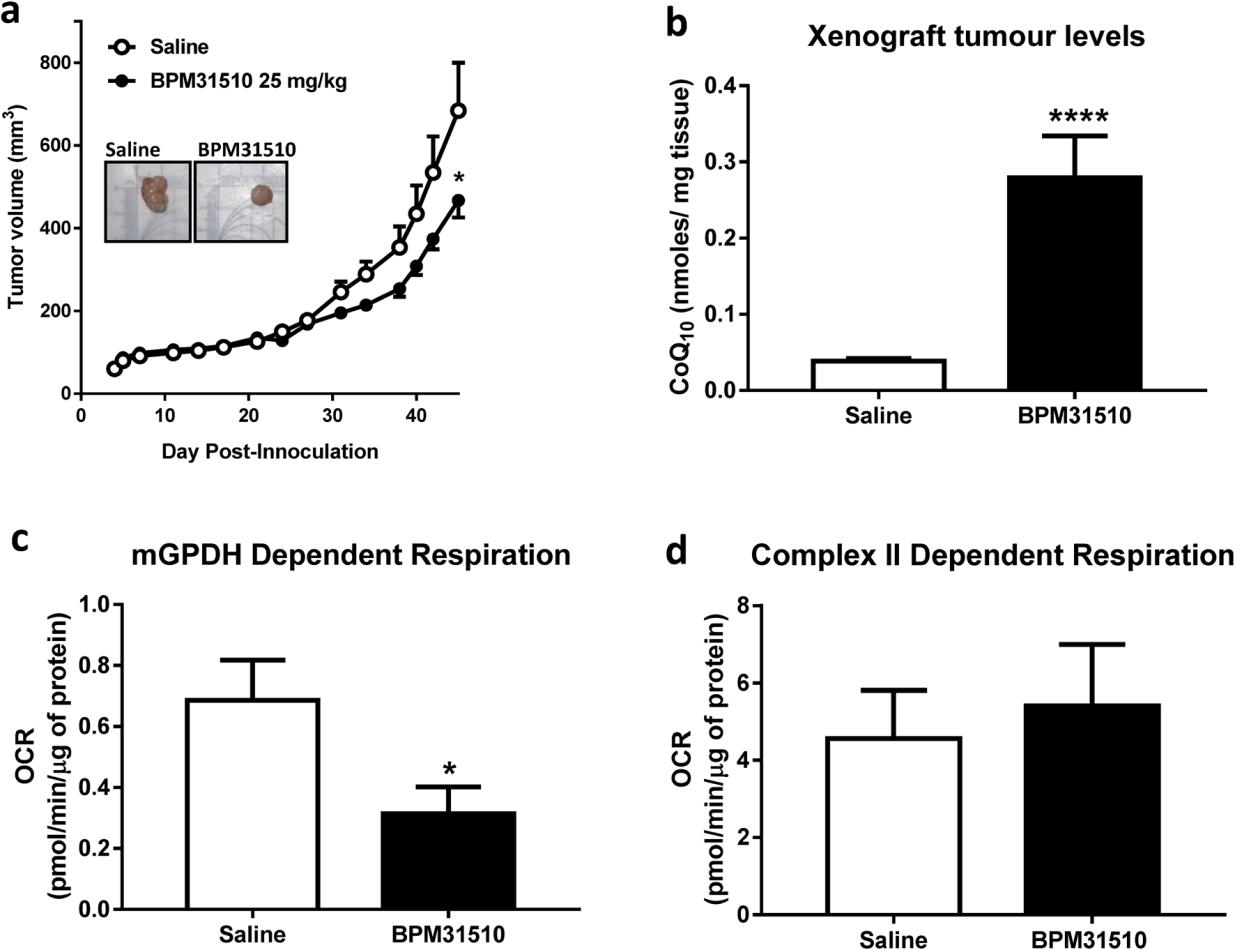
BPM31510 affects tumour size and alters mitochondrial respiration in vivo in a MIA PaCa-2 xenograft mouse model of pancreatic cancer. (**a**) BPM31510 (25 mg/kg, i.p., B.I.D.) effects on tumour volume in an MIA PaCa-2 xenograft pancreatic mouse model. Representative tumours from an untreated control and BPM31510-treated mouse are shown (inset). The data were analysed by Student’s t test; *p < 0.05 compared to control mice. (**b**) CoQ_10_ levels in tumours after BPM31510 treatment. Data represent n = 11 saline-treated mice and n = 12 BPM31510-treated mice. Data were analysed by Student’s t test. *p < 0.05 vs control. (**c**,**d**) BPM31510 effects on (**c**) state 3 G3PDH respiration and **(d)** state 3 Complex II respiration in MIA PaCa-2-derived epithelial cancer cells enriched from excised xenografts. N = 6 per group. The data represent the means + SEM and were analysed by a Mann–Whitney non-parametric test; ****p < 0.0001 compared to saline.

Next, the effects of delivering ubidecarenone by BPM31510 in vivo in an MIA PaCa-2 xenograft mouse model was determined. Intraperitoneal administration of BPM31510 (25 mg/kg, b.i.d) resulted in a significant decrease in tumour growth by day 45 after inoculation compared to saline-treated mice (Fig. 6a). In addition, CoQ_10_ tumour levels increased by six-fold over endogenous levels, indicating effective delivery of CoQ_10_ by BPM31510 after systemic administration (Fig. 6b). Notably, epithelial-enriched cancer cells (Supplemental Fig. 3) isolated from tumours of BPM31510-treated animals exhibited significantly decreased glyercol-3-phosphate-driven respiration, but not succinate-driven respiration, compared to saline-treated animals (Fig. 6c,d). These findings demonstrate that supraphysiologic increases in ubidecarenone levels have anti-cancer effects in vivo via site-selective disruption of mitochondrial respiration.

### Ubidecarenone decreases cell viability in patient-derived organoids and promotes substrate-specific ROS production

**Figure 7:**
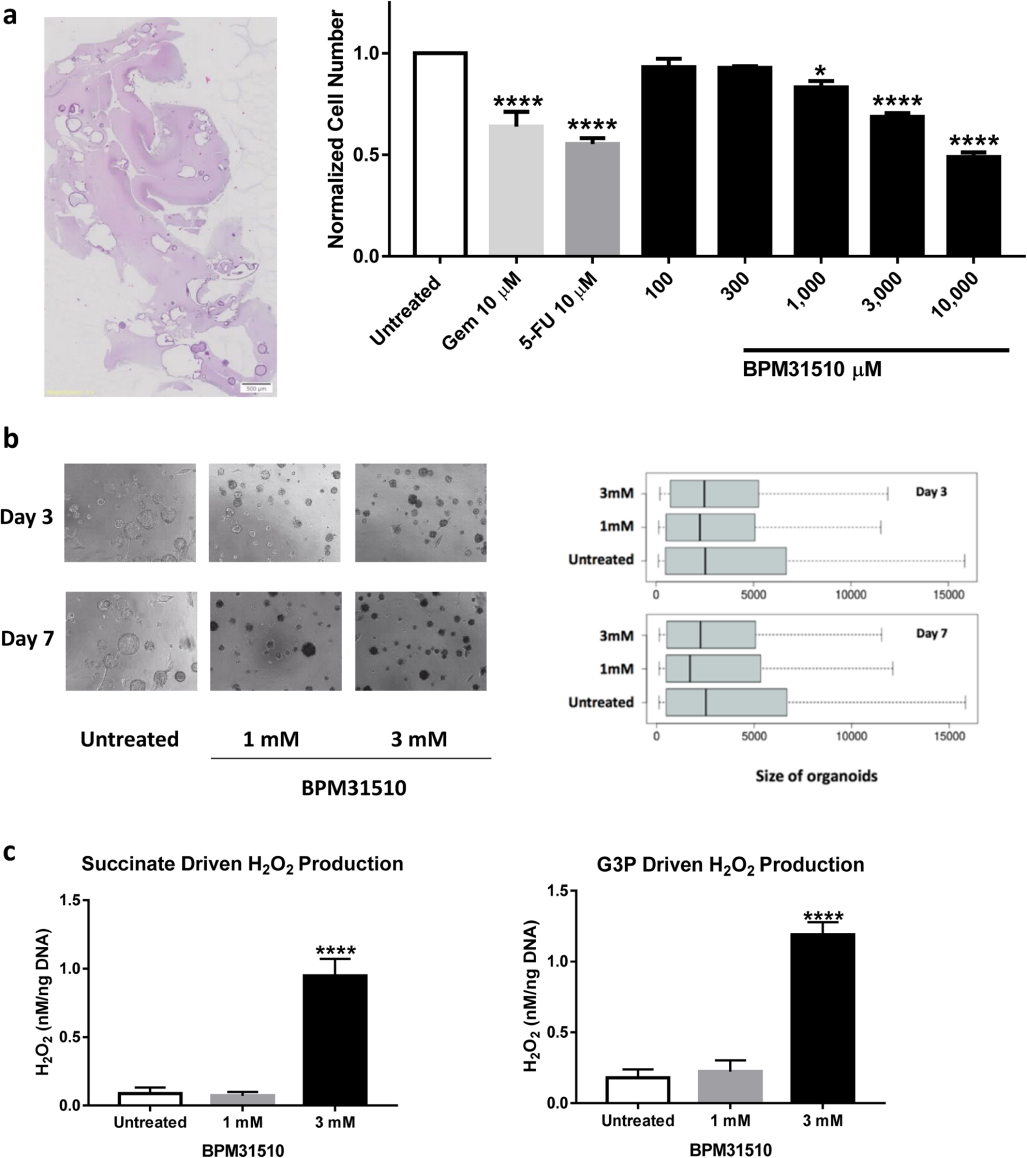
BPM31510 decreases cell viability in patient-derived organoids (PDO) derived from pancreatic tumours, alters G3PDH and Complex II-dependent respiration, and promotes substrate-specific reactive oxygen species (ROS) production. (**a**) Haematoxylin and eosin-stained PDOs (left panel). PDO viability after treatment with BPM31510 (right panel). PDOs were untreated or treated with either DMSO or a toxic mix of cyclohexane (50 µg/mL) and puromycin (2 µg/mL), gemcitabine (1 or 10 µM), 5-FU (1 or 10 µM), or BPM31510 (100–10,000 µM) for 72 h, and cell viability was assessed using a CytoTOX Glo cytotoxicity assay. The data represent the means ± SEM of three biological replicates. (**b**) BPM31510 decreases PDO size after 72 h of treatment. Representative images of PDO after 3 and 7 days of treatment with BPM31510 (left). The boxplots represent the distribution of PDO size following treatment (right). (**c**) Mitochondrial H_2_O_2_ generation during succinate-driven (left) and G3PDH-driven (right) respiration in PDOs after BPM31510 treatment. The data represent the means ± SEM of three independent experiments. All data were analysed by one-way ANOVA followed by Dunnett’s or Tukey’s post hoc test; *p < 0.05, ***p < 0.01, ****p < 0.001 compared to the untreated control group.

Due to limitations of cultured cell lines in vitro and cell line-derived xenograft models in vivo as an adequate representation of human disease pathophysiology, the effects of ubidecarenone was assessed in a primary ex vivo model using patient-derived pancreatic tumour organoids (PDO)^20^. Specifically, the effect of BPM31510 treatment was assessed in organoids derived from a male patient with stage 3 primary pancreatic adenocarcinoma. The organoids were assessed initially for maintenance of morphology, as assessed by haematoxylin and eosin staining (Fig. 7a). Consistent with the in vitro and in vivo models, BPM31510 induced a dose-dependent decrease in cell viability up 49% at the highest dose of 1 mM (Fig. 7a). Interestingly, BPM31510 treatment promoted changes in PDO size, as indicated by an increase in the appearance of smaller and medium-sized PDOs and a decrease in larger PDOs at days 3 and 7 after treatment (Fig. 7b). Importantly, BPM31510 treatment significantly enhanced H_2_O_2_ production in a succinate-dependent and G3P-dependent manner in this ex vivo model (Fig. 7c). These data demonstrate that the cellular effects of BPM31510 (via increasing mitochondrial ubidecarenone levels) are readily translated from primary preclinical in vitro models to human derived surrogate models of disease.

## Discussion

**Figure 8:**
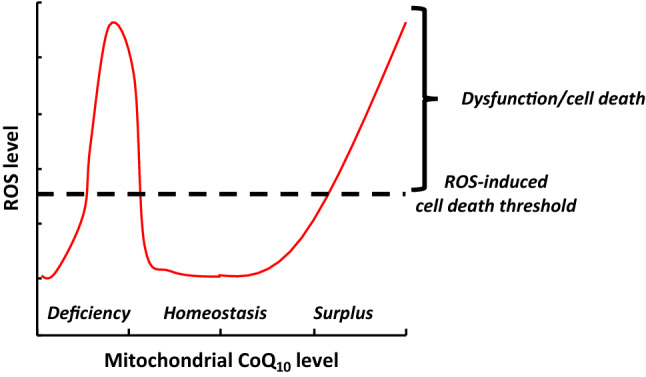
Relationship between mitochondrial CoQ_10_ level, reactive oxygen species (ROS) level, and ROS-induced cell death threshold. Both CoQ_10_ deficiency^22^ and CoQ_10_ surplus increase ROS levels, resulting in mitochondrial dysfunction and cell death, while homeostatic levels of CoQ_10_ support cellular function and survival. The ROS-induced cell death threshold likely differs between cell types.

This study demonstrates that mitochondrial delivery of supraphysiologic concentrations of oxidized CoQ_10_ using BPM31510 formulation disrupts ETC processes leading to significant increases in ROS generation. The increase in ROS leads to induction of apoptosis in pancreatic cancer, which has been consistently reproduced and translated from in vitro models through animal xenografts and human derived organoids. The study highlights the therapeutically under-leveraged concept of the anti-cancer potential of ROS induction beyond physiological threshold to activate cell death pathway in cancer^2^. Findings from the current study validate the argument that if the ROS levels in cancer cells are close to a threshold and are pushed over that threshold via a therapeutic modality, apoptosis can be triggered. Remarkably, normal cells with intrinsic lower levels of ROS compared to cancer cells would be spared the same fate of undergoing apoptosis, ensuring potential safety profile of the therapeutic modality^21^. Integrating these findings with the known molecular and biological consequences of CoQ_10_ and its deficiency syndromes^22^, it can be concluded that supraphysiologic levels of CoQ_10_ in the mitochondria disrupts redox homeostasis, activating cell death pathways in cancer while physiological CoQ_10_ levels maintain mitochondrial function and have potential to promote pro-survival responses and cellular growth (Fig. 8). Indeed, the safety and tolerability of BPM31510 has been established in Phase 1 clinical trial of solid tumors and is currently in clinical development for pancreatic cancer (NCT02650804), and in glioma (NCT03020602).

In this study, BPM31510 was used to delivery high concentrations of CoQ_10,_ specifically within mitochondria in pancreatic cancer models. Specifically, the observation in vitro in pancreatic cancer cells of an 80-fold increase in mitochondrial levels of ubidecarenone compared to 0.2–0.3 nmol/mg concentration observed in untreated cells highlights the impact of BPM31510 formulation in CoQ10 bioavailability. Few studies have been able to achieve high CoQ_10_ concentrations in mitochondria. Using a water-soluble formulation of CoQ_10_ known as Qter, Bergamini et al. achieved a CoQ_10_ concentration > 10 nmol/mg in mitochondria after treating T67 glioma cells with 10 µM Qter^23^. And, notably, co-administration of Qter with rotenone, a complex I inhibitor, appeared to potentiate rotenone-induced ROS generation compared to rotenone alone. The current study demonstrates that ubidecarenone induces an increase in ROS in a site-specific manner leading to disruption in mitochondrial respiration and activation of cell death.

Moreover, we observed a decrease in oxygen consumption rates in response to ubidecarenone delivery to the mitochondria. The data also demonstrated that the loss of electron transfer activity is coordinated with site-selective enhanced mitochondrial electron leak and subsequent ROS production. In the pancreatic cancer cells tested, delivery of supraphysiologic levels of ubidecarenone specifically affected succinate (Complex II) and glycerol-3-phosphate (GPDH)-fuelled respiration, with non-inhibitory effects on pyruvate (Complex I)-fuelled respiration. This functional impairment in respiration correlated with substrate-specific H_2_O_2_ production. Prior studies have indicated that succinate-driven H_2_O_2_ is mediated via reverse electron transport (RET) when the proton motive force (Δp) is high^24^.possibly due to an increase in levels of reduced CoQ_10_^9,25^. Of interest, Guarás et al. demonstrated that the balance of the CoQH_2_:CoQ ratio serves as a sensor for respiratory chain efficiency and that an increase in this ratio underlies ROS generation via RET^26^. In the present study, increasing the mitochondrial Q pool with BPM31510 increased succinate-mediated ROS production, which was detected in the presence of rotenone. Since this effect was not sensitive to rotenone in this study, it is unlikely that increasing CoQ_10_ promotes ROS via RET.

While CoQ_10_ serves as the electron transfer molecule for Complex I/III and Complex II/III-mediated respiration, the disruption of Complex II and GPDH-fuelled respiration that was observed is consistent with the known participation of Complex I in super-complexes with other ETC components^27^. Thus, RET may be mediated by a separate Q pool. Therefore, the observation that inhibiting Complex II and GPDH-fuelled respiration and promoting ROS production was elicited by exogenous supraphysiologic levels of ubidecarenone suggests an alteration in the CoQ_10_ pool that does not involve supercomplex-mediated electron transfer/respiration. This finding suggests that delivering CoQ_10_ into cancer cells with low levels of super-complexes and elevated FAD-oxidation and/or into highly metabolic tumours with increased TCA flux may be more susceptible to BPM31510-mediated pro-oxidant production and cell death. Such tumour subsets have been previously reported. For example, in a study of > 2000 breast cancer samples, Whitaker-Menezes et al. demonstrated transcriptional upregulation of oxidative phosphorylation genes and, specifically, increases in Complex II subunits/activity^28^. That sample indicates a tumour subset with elevated FAD oxidation amenable to recapitulation of the CoQ_10_ effects observed in this study.

In the pancreatic cell models tested in the current study, State 3 respiration on glyercol-3-phosphate was 2.5–4.4 pmol/min/µg protein, comparable to respiratory rates on pyruvate, succinate, and ascorbate/TMPD. In addition, even in the presence of multiple oxidizable substrates, GPDH-fuelled respiration accounted for 18–25% of total mitochondrial respiration. The effect of increased ubidecarenone on GPDH-fuelled respiration was more extensive compared to Complex II responses in cells isolated from the tumours of treated mice. The importance of GPDH was further substantiated from experiments in Rho0 cells, which showed greater sensitivity to ubidecarenone than their parental counterparts. Of particular interest, increased activity of mGPDH (G3PD) has been found in various tumours^29^. In addition, mGPDH links cytosolic glycolysis to mitochondrial ETC function via the NAD:NADH redox couple. Given that a function of glycolysis is consistent with the control of redox homeostasis, the data is consistent with prior studies that demonstrated that glyercol-3-phosphate can be a major contributor of substrate-fuelled mitochondrial ROS production^29^. However, while G3P/ROS has been described and G3P coordinates metabolic pathways important in cancer, it has not been considered as a relevant metabolic substrate in cancer to date. Results in this study emphasizes the underappreciated role for glyercol-3-phosphate as a substrate for mitochondrial respiration in cancer. As cancer is a disease in which metabolic substrate utilization is a distinct driver of disease progression and an emerging therapeutic target^18,30^, dependence on glyercol-3-phosphate as a metabolic substrate and subsequent ROS production may represent a ground-breaking anticancer target to be exploited in certain tumour types.

Furthermore, in this study the supraphysiologic effects of ubidecarenone were recapitulated in multiple model systems (in vitro, in vivo, ex vivo PDO), albeit to varying magnitudes. It is important to take note of a few limitations in the present study. The study demonstrates that Complex II and GPDH are sufficient for BPM31510-mediated ROS production/cell death. However, this does not negate the critical attributes to their absence in ubidecarenone effect. This is limited by the lack of genetic approaches to selective knockdown a mitochondrial complex or the availability of suitable compounds for site-selective pharmacological approach to prevent electron loading into the Q pool by GPDH. As such, one cannot determine the relative contributions of Complex II compared to GPDH. While mathematical models have been developed to address these important questions, they are theoretical and predicated on assumptions^24^. In addition, the findings reported here do not describe the effects of BPM31510 on comparative healthy/non-tumorigenic cells. However, supraphysiologic concentrations achieved in clinical trials of BPM31510 are associated with reliable and consistent safety and tolerability profiles (NCT02650804, NCT03020602, NCT01957735), supporting non-deleterious effects in “normal” cells compared to cancer.

In summary, the data demonstrates the potential of an anti-cancer therapeutic modality that builds upon early strategies that have attempted to disrupt redox homeostasis. In addition, the study provides evidence that the mitochondrial resident molecule has the ability to impact cancer outcomes by influencing key mitochondrial function. Early approaches aimed to promote antioxidant activity for anti-cancer effect targeted antioxidants and ROS-scavenging systems, such as superoxide dismutase (SOD), glutathione peroxidases, peroxidoxins, glutaredoxin, thioredoxin, catalase, and nuclear factor erythroid 2-related factor 2 (Nrf2). Unfortunately, antioxidant treatments have been ineffective^31^. The data in this study clearly demonstrate that a pro-oxidant state induced by enhancing pro-oxidant production is essential for an effective anti-cancer strateg^17,32^. This study successfully leveraged existing and emerging science to establish the ability of BPM31510 to deliver supraphysiologic levels of CoQ_10_ to the mitochondria offers a distinct, novel, safe, and potentially effective therapeutic strategy aimed at targeting ROS, oxidative stress, and activation of cell death pathways in the treatment cancer.

## Methods

### Antibodies and chemical reagents

The following antibodies were used in this study: Annexin V FITC-conjugated (Molecular Probes), mouse fibroblast specific protein-1 (Abcam), mouse CD31 PE-conjugated (Militenyi), mouse CD14 PerCP Cy5.5-labelled (BD Biosciences), mouse B220 PE-Cy5-labelled (BD Biosciences), and human CD44 APC-conjugated (Militenyi) antibodies. The goat anti-mouse IgG Alexa 488 secondary antibody was obtained from Invitrogen. The BPM31510 formulation is a colloidal nanodispersion mixture containing CoQ_10_ in combination with dimyristoylphosphatidylcholine (DMPC) and the surfactant poloxamer 188 in a fixed ratio that renders a relatively stable formulation. To produce BPM31510, CoQ_10_ was mixed with sterile water and placed in a water bath at ~ 65 °C to melt. The melted CoQ_10_/water mixture was mixed with DMPC and poloxamer 188 with constant stirring with a polytron homogeniser to form dispersed particles. The mixture was then subjected to particle size reduction in a microfluidizer. The particle size distribution was measured after successive cycles of the microfluidization process using a particle size analyser until the mean particle size of the formulation was < 200 nm.

### Pancreatic cancer cell lines

The human pancreatic adenocarcinoma cell lines MIA PaCa-2 and PANC1 were obtained from Sigma-Aldrich and cultured at 37 °C with 5% CO_2_ in Dulbecco’s modified Eagle’s medium (DMEM, Thermo Scientific) supplemented with 5% foetal bovine serum (FBS) (Gibco) and 1% penicillin–streptomycin-amphotericin A (Lonza). Experimental procedures were performed within 20 passages after thawing, and pancreatic cancer cells were discarded after 30 passages in culture.

### Cytotoxicity assays

Cells were plated at a density of 1.0 × 10^4^ cells per well in 96-well black plates and treated with BPM31510 (0–12,800 µM). Cells were incubated for 72 h, and relative cell viability was determined based on relative protease activity using CellTiter-Fluor viability reagent (Promega) according to the manufacturer’s instructions. Briefly, the cell culture medium was replaced with DMEM without phenol red supplemented with d-glucose, sodium pyruvate, Glutamax, sodium bicarbonate, and the fluorogenic, cell-permeant peptide substrate Gly-Phe-AFC (1:2000, Promega). Cells were incubated for 1 h at 37 °C, and cleavage of the substrate by active cell proteases was fluorometrically measured at an excitation of 390 nm and emission of 505 nm. The BPM31510 half maximal effective concentration (EC_50_) and EC_90_ 90% effective concentration (EC_90_) values for each cell line were determined by nonlinear regression analysis in GraphPad Prism.

### Clonogenic survival assay

Cells were plated at a density of 1.0 × 10^5^ cells per well in 12-well plates in growth media and then treated with BPM31510 at the indicated doses for 72 h at 37 °C. The cells were then trypsinized and counted, and 1,000 cells from each treatment group were re-plated in 6-well plates in growth media without drug treatment for 10 or 12 days for MIA PaCa-2 or PANC1 cells, respectively. The growth media were aspirated from each well and the wells were washed in PBS before the cells were fixed in 70% ethanol for 5 min. To visualize colony formation, plates were counterstained with 1% crystal violet for 10 min, and the number of colonies (> 50 cells) was manually counted per treatment group.

### Apoptosis measurements

Cells were plated at a density of 1.0 × 10^5^ cells in 24-well dishes and treated with BPM31510 at the doses and times indicated. The cell culture supernatant (containing detached apoptotic cells) and the adherent cells were harvested by trypsinization and centrifuged at 500×*g* for 5 min, before the resulting pellets were washed in staining media (PBS, 0.5% FBS). The cells were co-stained with FITC-conjugated anti-Annexin-V (1:200, Molecular Probes) to detect phosphatidylserine (PS) exposure on the outer membrane surface, and with propidium iodine (PI, 1:2000, Molecular Probes) in 200 μL binding buffer (Molecular Probes). After a 15-min incubation in the dark, the percentage of Annexin V^pos^ and PI^pos^ cells was analysed using the FL-1 and FL-3 channels on an Accuri C6 Flow Cytometer (BD Biosciences).

### Mitochondrial membrane potential

Cells were plated at a density of 1.0 × 10^5^ cells in a 12-well dish and treated with BPM31510. At 48 and 72 h, the cells were stained with 200 nM tetramethylrhodamine ethyl ester (TMRE; Abcam) for 20 min at 37 °C. The cells were then washed twice in PBS, trypsinized, and stained with Annexin-V and PI to gate apoptotic cells. The sequestration of TMRE by polarized mitochondria was analysed in the FL-2 channel using flow cytometry.

### Coenzyme Q_10_ imaging and quantitation

#### Confocal imaging

A BPM31510 formulation containing a fluorescent analogue closely resembling the CoQ_10_ structure was designed. The probe consists of three segments: a phenol head group (CoQ_10_-like head group), a reporter (fluorescent dye), and a lipophilic segment (9 units isoprenyl group). First, a solanesyl-α-formyl pyrrole adduct was prepared and then assembled to the asymmetric BODIPY containing the solanesyl isoprenyl group. This was followed by the preparation of the dimethoxy phenol head group. The last step of the synthesis relied on Knoevenagel condensation to couple the two segments. Finally, the formulation was prepared containing 4% CoQ10 analogue, 3% DMPC, and 1.5% Poloxmer 188. The cells were treated with 200 µM of the formulation for 24 h or 48 h and costained with MitoTracker Green (Molecular Probes). Live cell images were captured using a confocal microscope (Olympus IX83) at excitation at 488 nm for MitoTracker Green and 543 nm for the red fluorescent CoQ_10_.

#### Quantitation of subcellular CoQ_10_ distribution by MS/MS^all^

Cells were plated at a density of 1.0 × 10^6^ cells/well and treated with BPM31510 at the indicated doses and times. The cells were then washed once in ice-cold PBS, trypsinized, pelleted, and then homogenized in mitochondrial isolation buffer (0.21 M mannitol, 0.07 M sucrose, 0.1 mM EDTA, 1 mM EGTA, 10 mM Tris HCl, 0.5% BSA, pH 7.4 using KOH). Mitochondrial, cytosolic and nuclear/plasma membrane fractions were obtained by sucrose gradient centrifugation. To quantify the accumulation of BPM31510 in the subcellular fractions, an automated structural lipidomics platform was utilized as previously described^33^. Briefly, to extract lipids, 4 mL CHCl_3_/MeOH (1:1, v:v) was added to each sample tube and vortexed for 20 min. Another 2 mL of 50 mM LiCl was added, and the samples were mixed for 10 min and centrifuged at 2000 rpm for 5 min. The bottom chloroform layer was then extracted, and 1.8 mL of CHCl_3_ was added to the source tubes. Samples were mixed for 5 min and centrifuged again at 2000 rpm for an additional 5 min. The extraction method was repeated two more times prior to drying under nitrogen and reconstituted and loaded onto a SCIEX 5600 + TripleTOF. CoQ_10_ measurements were quantitatively measured based on a CoQ_10_ standard curve.

### Mitochondrial complex activity in permeabilized cells

To test mitochondrial ETC complex activity in pancreatic cancer cells, 1.0 × 10^4^ cells/well were plated in specialized plates (Agilent Technologies) in the corresponding growth media and incubated for 3 h prior to treatment with BPM31510. At 18 h post-treatment, the cells were washed once in mitochondrial assay solution (MAS; 220 mM mannitol, 70 mM sucrose, 20 nM H_2_KPO_4_, 5 mM MgCl_2_, 2 mM HEPES, 1 mM EGTA, pH 7.4) and immediately reconstituted with XF plasma membrane permeabilization buffer (1 nM XF-PMP, 4 mM ADP, MAS buffer, Seahorse Biosciences). Complex I-, II-, and IV-driven respiration was assessed by electron flow assay (Seahorse Biosciences). Briefly, the cells were treated with 10 mM pyruvate and 1 mM malate (Complex I) followed by sequential treatment with rotenone (2 µM), succinate (Complex (II), 10 mM), Antimycin A (1 µM), and ascorbate (10 mM) + TMPD (N,N,N′,N′-tetramethyl-p-phenylenediamine, 100 µM). To measure GPDH-driven respiration, the cells were seeded at a density of 1.0 × 10^4^ cells per well and treated with BPM31510. At 18 h post-treatment, the cells were washed once in mitochondrial assay solution and immediately reconstituted with XF plasma membrane permeabilization buffer supplemented with 5 mM glycerol-3-phosphate and 2 µM rotenone. Non-mitochondrial oxygen consumption rate (OCR) was determined by injection with Antimycin A and rotenone, and residual oxygen consumption was subtracted from the results to obtain mitochondrial-specific rates. Total mitochondrial oxidation and complex-dependent OCR levels were measured as described, with modifications. After 18 h, the cells were washed once in 1× MAS buffer and immediately reconstituted with XF PMP buffer (1 nM XF PMP, 4 mM ADP, 1× MAS buffer, and a combination of pyruvate, malate, succinate, and G3P. To determine complex dependency, rotenone (1 µM final, Complex I inhibitor), TTFA (250 µM, Complex II inhibitor) and i-GP1 (400 µM, mGPDH inhibitor) were sequentially injected in different combinations. Complex dependency was determined by measuring the decrease in OCR in the presence of inhibitors for the targeted complex expressed as the percentage of oxidation of all three oxidizable substrates. All the data were normalized to total protein content measured using separate plates set up in parallel.

### ROS measurements

Approximately 1 × 10^5^ cells were seeded in 12-well dishes and treated with BPM31510. After 24 h, the cells were washed in PBS and incubated with 5 µM *CM*-*H*_*2*_*DCFDA* (Life Technologies), 2.5 µM CellRox Red (Thermo Fisher), or 2.5 µM CellRox Green (Thermo Fisher) for 30 min prior to analysis by flow cytometry. All samples were costained with PI to exclude the dead cell population from the analysis. Complex-driven H_2_O_2_ production was measured using Amplex Red (Thermo Fisher), according to the manufacturer’s instructions but with the following modifications: after 24 h of BPM31510 treatment, the media were aspirated and the cells were washed with 1× MAS buffer. To measure complex-specific H_2_O_2_ production, the cells were incubated with 10 mM pyruvate/1 mM malate, 10 mM succinate/2 µM rotenone, or 5 mM glycrol-3-phosphate/2 µM rotenone diluted in MAS buffer containing 4 mM ADP and 1 nM XF PMP for 30 min at 37 °C prior to the addition of Amplex Red. H_2_O_2_ production was kinetically monitored for 90 min at 37 °C using an excitation of 530 and emission of 590. Total H_2_O_2_ production was quantitated based on a H_2_O_2_ standard curve (Supplemental Fig. 1).

### Generation of Rho0 Cells

Mitochondria were depleted by culturing MIA PaCa-2 cells in the presence of 100 ng/mL ethidium bromide for 6–8 weeks. The growth culture media were supplemented with 4.5 mg/mL glucose, 50 μg/mL uridine, and 100 μg/mL pyruvate to compensate for respiratory metabolism defects. After establishing Rho0 cells, the media was supplemented with 50 μg/mL uridine for cell growth and expansion. To validate the successful generation of Rho0 cells, OCR and extracellular acidification rate (ECAR) levels were tested using a Seahorse XF96 Analyzer (Agilent).

### In vivo xenograft studies and cancer cell extraction

#### Animals

All animal experiments were conducted in compliance with the relevant laws and institutional guidelines of the Charles River Laboratory. Seven-week-old athymic nude mice from Charles River Laboratory were inoculated with MIA PaCa-2 tumour cells (ECACC). Each mouse was inoculated subcutaneously on the right flank with MIA PaCa-2 tumour cells (5 × 10^6^) in 100 µL of Dulbecco's modified Eagle’s medium with 50% Matrigel (Corning Catalogue No. 47743-715) for tumour development. Two days after tumour cell inoculation, the mice were assigned to one of two groups consisting of 18 animals each to receive either vehicle (saline) or 25 mg/kg BPM31510 intraperitoneally in a volume of 10 mg/kg. Treatment continued for 47 days until the largest tumour in the control group reached 2000 mm^3^. Tumour dimensions and body weights were recorded three times per week with a gap of 2–4 days between the two measurements. The tumour volume was calculated as 0.5 × Long Diameter × Short Diameter.

#### Cancer cell extraction

The protocol for extraction of the enriched cancer cell population was based on previously described methods^34^. Briefly, tumour xenografts were minced into small mm^3^ pieces and digested in collagenase (850 units/mL collagenase type II, 2.5 mg/mL trypsin, 5 mg/mL albumin resuspended in PBS) for 20 min. After 20 min, the collagenase solution was removed by straining samples through a 100 µM cell strainer, and tumour tissues were again digested for an additional 20 min. The single cell suspension was then collected, and the samples were incubated for 20 min to allow for the epithelial cancer cells and fibroblasts to separate (as per the sedimentation technique described by Lanari et al.^30^). After incubation, 10 mL cell media was removed, generating the first wash. The washing steps were repeated a further four times. After the final wash, all tubes were centrifuged at 250×*g* for 10 min, and the supernatant was removed. Cells were resuspended in growth media and counted using trypan blue exclusion to determine the percentage of viable cells prior to mitochondrial measurements. Complex II (succinate) and GPDH-driven respiration was measured as described above. Briefly, 100 µL of cell suspension containing approximately 300,000 cells was plated per well in technical replicates. The plates were then spun for 20 min at 4 °C at 2200 rpm. The media was removed, and the plates were washed in 1× MAS buffer to remove residual media. The plates were then replenished with 10 mM pyruvate + 1 mM malate or 5 mM glycerol-3-phsophate + 2 µM rotenone in 1× MAS buffer supplemented with 4 mM ADP and 1 nM XF-PMP. An additional plate was set up in parallel to determine protein levels. Cancer cell enrichment was measured using the following cell surface expression markers: fibroblast specific protein-1 (mouse 1:400, secondary goat anti-mouse IgG Alexa 488 1:2000), CD31 (mouse PE-conjugated, 1:200), CD14 (mouse PerCP Cy5.5, 1:200), B220 (mouse PE-Cy5, 1:200), and CD44 (human, APC conjugated), and the percentage of CD44^pos^ cancer cells was measured by flow cytometry (Supplemental Fig. 2).

### Generation of patient-derived organoids

Patient-derived tumour organoid (PDO) cultures were generated from a primary pancreatic adenocarcinoma tumour obtained from a Caucasian male patient (age 69) with Stage 3 cancer and no evidence of local or distant metastasis. The patient harboured the following genetic point mutations: *KRAS* (G61H), *SMAD4* (Asn107Lys), *BRCA2* (Arg2034Cys), and *PRSS* (Cys171Tyr). The following screened genes were normal: *GNAS*, *BRAF*, *FGFR1*, *MYC*, *MDM2*, *GATA6*, *AKT2*, *TP53*, *CDKN2A*, *RNF43*, *RREB1*, *BRCA1*, *ATM*, *PALB2*, *ARID1A*, *PBRM1*, *MLL3*, *MLL4*, *KDM6A*, and *NF1*. The generation of the PDO was performed as described previously^20^. Briefly, tissue from tumour resections was minced into DMEM containing 1% of each BSA and penicillin/streptomycin. The digested tissue was plated using specialized media developed for organoid development into a single well of a 12-well plate coated with Matrigel. After 10–15 days to allow for generation, the organoids were cultured and maintained at 37 °C in 5% CO_2_.

Organoids from a 12-well plate were first seeded into a Matrigel-coated 96-well assay plate (Corning) in pancreatic tumour organoid development media at a density of 5.0 × 10^4^ cells/well (day 0). On day 4, PDOs were treated with varying concentrations of BPM31510, gemcitabine, or DMSO control using a TECAN D300e auto drug dispenser and kept at 37 °C for 4 days in a CO_2_ incubator. Each drug concentration was added into three different wells per organoid line, and three independent trials were performed that produced nine different replicates. On day 8, cell survival was analysed using a CytoTOX Glo cytotoxicity/cell survival assay (Promega) following the manufacturer’s instructions, using a BioTek synergy H1 microplate reader. Data were finalized by normalizing the cell survival in drug-treated wells with respect to the carrier control.

### PDO image analysis

For analysis of PDO size, images were captured under a light microscope and processed and quantified using Open Source Computer Vision Library (https://opencv.org/). The images were processed using a python script (Supplemental Fig. 3) that automatically detects and segments PDOs from the background. Specifically, images were first converted to greyscale and then Gaussian blurred to fill breaks in the organoid edges, and the edges were detected by a Canny edge filter. The edges were Gaussian blurred to start the smearing. The smearing was further processed with a morphologically close operation. Small noise particles were denoised with a morphological open operation. Unsegmented bubbles in the middle of the organoids, especially in organoids with dark black centres, were flood filled to complete the segmentations. A connected component filter was used to count and measure the pixel areas of all segmented organoids. The mean intensities of each organoid were calculated by taking the intensities from the original images using the PDO segmentation images to obtain the pixel locations.

After segmenting the organoids, the size of organoids was defined by pixel areas. The pixel areas of all organoids were plotted in a histogram to determine the signal from noise. The area histogram identified mostly smaller areas with a long sparse tail, indicative of large areas. Areas after the contiguous tail were considered noise and excluded from the analysis by thresholding objects above 40,000 pixels in area for the area analysis. At this point in the analysis, information for each organoid, treatment, area and time point was retained.

### Statistics and reproducibility

Data are presented as the means ± standard error of the mean from 3 to 5 biological replicates with least three independent experiments for cell and PDO models. Statistical testing was performed using two-tailed t-tests for comparisons between two groups and ANOVA for comparisons among three or more groups. Post hoc tests for multiple comparisons are specified in the figure legends. For data that were not normally distributed, non-parametric tests were used and described in the figure legends. To test for the effect of treatment on PDO size, organoids treated with 1 mM or 3 mM BPM31510 were labelled as ‘treated’, while organoids that were not treated were labelled ‘untreated’. Regression models (‘PDO size’ × ‘Treatment’) were built in R for each time point to determine the relationship between treatment and PDO size. The detailed linear regression models are shown in Supplemental Fig. 4. A *p* < 0.05 was considered statistically significant. All statistical analyses were performed using GraphPad Prism version 7 (GraphPad).

## Supplementary information

Supplementary information.

## Data Availability

The data that support the findings of this study are not publicly available due to their propriety nature. Inquiries should be directed to the corresponding author [RS].
