# Supplementary material for: Elevated levels of mitochondrial CoQ10 induce ROS-mediated apoptosis in pancreatic cancer
Source: Sci Rep. 2021 Mar 11;11:5749. doi: 10.1038/s41598-021-84852-z (PMC7952582; doi:10.1038/s41598-021-84852-z)

**Title:**

Elevated levels of mitochondrial CoQ_10_ induce ROS-mediated apoptosis in pancreatic cancer

**Author Block:** Tulin Dadali^†1^, Anne R. Diers^†1^, Shiva Kazerounian^1^, Senthil K. Muthuswamy^2^, Pallavi Awate^1^, Ryan Ng^1^, Saie Mogre^1^, Carrie Spencer^1^, Katerina Krumova^1^, Hannah E. Rockwell^1^, Justice McDaniel^1^, Emily Y. Chen^1^, Fei Gao^1^, Karl T. Diedrich^1^, Vijetha Vemulapalli^1^, Leonardo O. Rodrigues^1^, Viatcheslav R. Akmaev^1^, Khampaseuth Thapa^1^, Manuel Hidalgo^2^, Arindam Bose^2^, Vivek K. Vishnudas^1^, A. James Moser^2^, Elder Granger^1^, Michael A. Kiebish^1^, Stephane Gesta^1^, Niven R. Narain^1^, Rangaprasad Sarangarajan*^1^

^†^Designates joint first authorship

^1^BERG LLC, Framingham, MA, 01701, USA

^2^Department of Medicine, Cancer Research Institute, Beth Israel Deaconess Medical Center, Harvard Medical School, Boston, MA, 02215, USA.

**Corresponding Author**

Rangaprasad Sarangarajan, Ph.D.

500 Old Connecticut Path, Bldg B, 3^rd^ Floor

Framingham, MA 01710

Email: [Rangaprasad.Sarangarajan@BergHealth.com](mailto:Rangaprasad.Sarangarajan@BergHealth.com)

Phone: 617-588-2164

**Supplemental Figures and Table Legends**

**Supplemental Table 1.** *Cellular delivery of CoQ_10_ by BPM31510 accumulates in the mitochondria of MIA PaCa-2 and PANC1 cells in a time-dependent manner.* Cells were treated with BPM31510 (EC_50_) for 24 or 48 h, washed, and lysed. Subcellular fractionation was carried out by sucrose gradient centrifugation. Total CoQ_10_ levels were measured by LC MS/MS. The data represent the means + SEM of total protein (nmol/mg) from six independent experiments. The data were analysed by one-way ANOVA followed by Tukey’s post hoc test.

**Supplemental Figure 1.** *Time-dependent substrate-specific driven H_2_O_2_ production using the Amplex® Red assay.* (**a**) Dose and time effects of menadione on pyruvate-driven H_2_O_2_ production assessed by modified Amplex® Red assay in MIA PaCa-2 pancreatic cancer cells. The H_2_O_2_ standard curve is shown in the inset. The data represent three independent experiments. (**b**) Dose effects of menadione on pyruvate-mediated H_2_O_2_ generation at 90 min post treatment. (**c-d**) Kinetic graphs illustrating substrate-specific H_2_O_2_ generation with or without BPM31510 treatment assessed over 90 min in (**c**) MIA PaCa-2 and (**d**) PANC1 cells. UD = undetected.

**Supplemental Figure 2.** *Tumour enrichment and substrate driven respiration.* (**a**) Enrichment of epithelial cancer cells from immunocompromised mice with MIA PaCa-2 xenografts. Representative scatter plots showing the gating strategy of fibroblast specific (FSP-1), CD44 and CD31-positive stained cells. (**b**) Table reporting the percentage of CD44-positive cells plated for subsequent Seahorse assays. Cells plated to measure substrate-driven respiration were predominantly from the epithelial-derived cancer cell population. (**c**) Complex II-dependent and (**d**) Complex IV-dependent respiration assessed in tumours from control and BPM31510-treated mice. The data represent mean + SEM of n=6 saline-treated (control) and n=5 BPM31510-treated mice.

**Supplemental Figure 3.** *PDO image processing source code for segmentation.*

**Supplemental Figure 4.** *Regression models to test for effect of treatment on PDO size.* A linear regression model was built for day 3 (left panel) and day 7 (right panel) time points using the glm() function from the base R library. The dose was modelled as a binary variable: ‘Dose – no treatment’ and ‘Dose – any treatment’. The models indicate that the PDOs tend to be larger when there is no treatment and that the difference is statistically significant based on their p-value.

**Supplemental Table 1**

|  | **24 h** | | | | **48 h** | | | |
| --- | --- | --- | --- | --- | --- | --- | --- | --- |
|  | **Untreated** | | **EC_50_** | | **Untreated** | | **EC_50_** | |
|  | **Mean (nmol/mg) ± SEM** | **% of Total** | **Mean (nmol/mg) ± SEM** | **% of Total** | **Mean (nmol/mg) ± SEM** | **% of Total** | **Mean (nmol/mg) ± SEM** | **% of Total** |
| **Cytosol** | | | | | | | | |
| **MIA PaCa-2** | 0.11 ± 0.03 | 18.0% | 5.17 ± 0.54^*^ | 9.7% | 0.19 ± 0.04 | 35.2% | 14.07 ± 2.54^****,+++^ | 8.5% |
| **PANC1** | 0.09 ± 0.01 | 15.2% | 4.06 ± 0.31^****^ | 7.9% | 0.08 ± 0.01 | 12.0% | 7.01 ± 0.55^****,++++^ | 6.3% |
| **Nucleus/Plasma membrane** | | | | | | | | |
| **MIA PaCa-2** | 0.18 ± 0.03 | 30.2% | 22.47 ± 2.97^*^ | 42.0% | 0.14 ± 0.02 | 26.2% | 62.99 ± 12.19^****,+++^ | 38.2% |
| **PANC1** | 0.21 ± 0.02 | 40.6% | 17.72 ± 2.03^*^ | 34.7% | 0.31 ± 0.04 | 41.3% | 39.60 ± 8.79^****,++^ | 35.5% |
| **Mitochondria** | | | | | | | | |
| **MIA PaCa-2** | 0.31 ± 0.13 | 51.8% | 25.90 ± 3.00^*^ | 48.4% | 0.21 ± 0.01 | 38.7% | 87.83 ± 14.06^****,++++^ | 53.3% |
| **PANC1** | 0.22 ± 0.21 | 44.1% | 29.34 ± 2.39^**^ | 57.4% | 0.35 ± 0.04 | 46.7% | 64.80 ± 10.73^****,+++^ | 58.2% |
| **** p<0.0001 vs. untreated; +, p<0.05, ++ p<0.01, and +++ p<0.001 vs. 24 hr EC_50_ | | | | | | | | |

**Supplemental Figure 1**


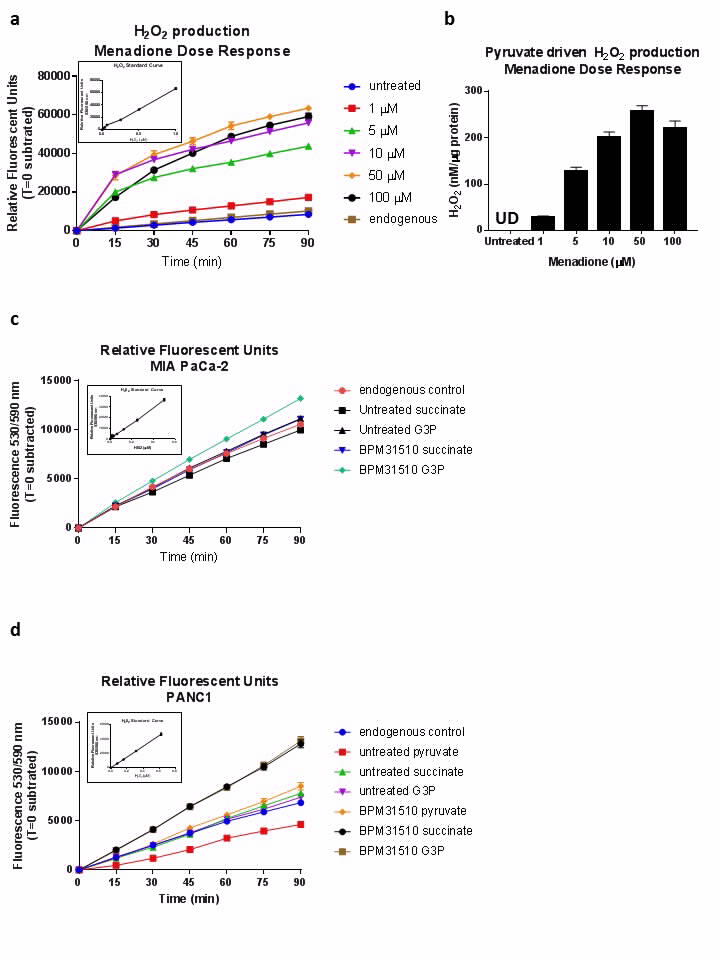


**Supplemental Figure 2**

**
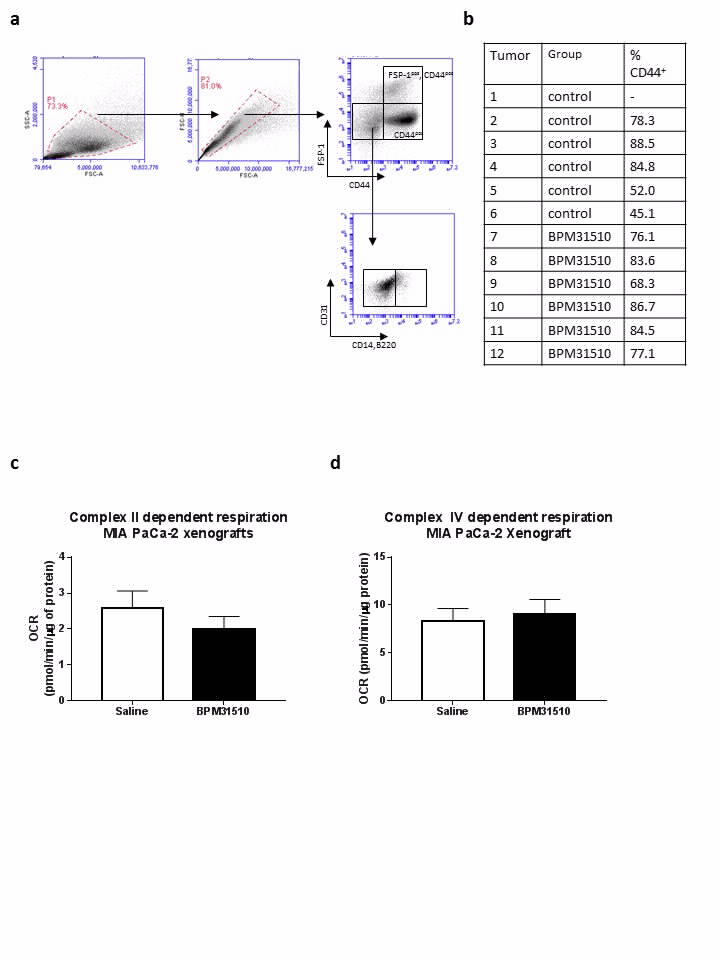
**

**Supplemental Figure 3**

**The Python Script for the image segmentation portion of the source code is:**

self._gray = cv2.cvtColor(self._img, cv2.COLOR_BGR2GRAY)

blurred = cv2.GaussianBlur(self._gray, (3, 3), 0)

edges = cv2.Canny(blurred, 25, 100, 7)

# blend edges together into objects

blurred_edges = cv2.GaussianBlur(edges, (7, 7), 0)

kernel = np.ones((6, 6), np.uint8)

blended_objects = cv2.morphologyEx(blurred_edges, cv2.MORPH_CLOSE, kernel, iterations=2)

# noise removal

kernel = np.ones((15, 15), np.uint8)

denoised_blends = cv2.morphologyEx(blended_objects, cv2.MORPH_OPEN, kernel, iterations=1)

filled_blends = fill_holes(denoised_blends)

fg_components = cv2.connectedComponentsWithStats(filled_blends, connectivity=self._connectivity)

**Supplemental Figure 4**


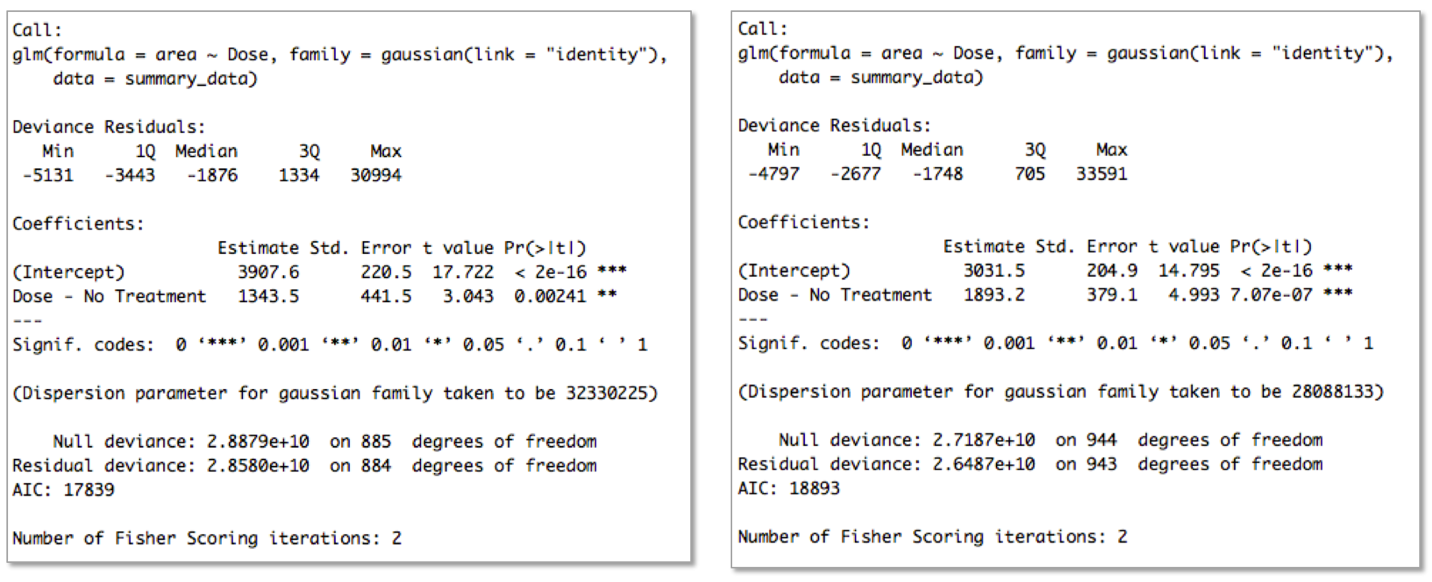

Supplement: Supplementary file 1 — Supplementary information. [file 41598_2021_84852_MOESM1_ESM.docx]
